# Poor insight and self-disorders in schizophrenia: an empirical study

**DOI:** 10.1007/s00406-025-02035-7

**Published:** 2025-06-03

**Authors:** Lars Siersbæk Nilsson, Julie Nordgaard, Mads Gram Henriksen, Josef Parnas, Andreas Rosén Rasmussen

**Affiliations:** 1 Mental Health Center Amager, Copenhagen University Hospital, Copenhagen, Denmark; 2 Institute of Clinical Medicine, Faculty of Health and Medical Sciences, University of Copenhagen, Copenhagen, Denmark; 3 Psychiatry East, Region Zealand, Roskilde, Denmark; 4 Center for Subjectivity Research, Department of Communication, University of Copenhagen, Copenhagen, Denmark; 5 Mental Health Center Glostrup, Copenhagen University Hospital, Copenhagen, Denmark; 6 Center for Youth Mental Health, The University of Melbourne, Melbourne, VIC Australia

**Keywords:** Phenomenology, Psychosis, EASE, Psychopathology, Subjective experience

## Abstract

Poor insight is a core feature of schizophrenia and empirical studies have demonstrated associations with important symptom domains, cognitive functions, and clinical outcomes. While recent explanatory accounts mostly focus on neuro-, social-, or meta-cognitive deficits, a complementary model from phenomenological psychopathology suggests that impaired insight in schizophrenia is tied to fundamental alterations to the structure of subjectivity, viz. self-disorders affecting the conditions for self-reflection, that usually precede psychotic experiences and persist temporally beyond state-psychopathology. In line with this account, we hypothesized that self-disorders would be associated with impaired insight independently of shared associations with other forms of psychopathology and general intelligence. A sample of 67 patients with a diagnosis of schizophrenia or non-affective psychosis in non-acute phase of illness underwent comprehensive psychopathological examination including the Examination of Anomalous Self-Experiences (EASE) for assessment of self-disorders and the Positive and Negative Syndrome Scale (PANSS). PANSS item G12 was used as measure of insight. The group with impaired insight was characterized by a significantly higher level of self-disorders than the group with good insight, but there were no significant between-group differences regarding positive, negative, or depressive symptoms. In simple linear regression analysis only self-disorders were significantly associated with impaired insight, and multiple linear regression showed similar results. Our findings support the theoretical claim that self-disorders are linked to poor insight in schizophrenia. If corroborated this may have implications for early intervention and treatment.

## Introduction

Compromised insight has traditionally been considered an integral feature of schizophrenia. Jaspers, for instance, noted that though patients with chronic psychotic manifestations might prima facie demonstrate good insight by employing certain clinical terms, this usually reflected an appropriation of psychiatric jargon and not a truly meaningful re-evaluation of the nature of their experiences [1] (p. 423 − 24). In recent decades, the development of various psychometric scales assessing insight into illness has facilitated a surge in empirical research interest [2]. As noted by Marková and Berrios, the dissimilarities of these instruments reflect important theoretical issues [3]. Over time, a binary view on insight as a largely all-or-nothing phenomenon has given way to more continuous and multi-dimensional approaches. While some instruments like the Positive and Negative Syndrome Scale (PANSS) employ a unidimensional or categorical approach [4], others like the Scale to Assess Unawareness of Mental Disorder (SUMD) treat insight as a multidimensional construct [5]. Another distinction is between scales like the aforementioned that gauge clinical insight, i.e., the awareness of having a mental disorder and symptoms, and complementary instruments targeting cognitive insight. i.e., the ability to cognitively evaluate one’s anomalous experiences and beliefs [6].

Notwithstanding these conceptual differences, there is evidence for associations between impaired insight and important clinical features and outcomes. On the symptomatic level, an influential meta-analysis demonstrated a negative relationship between insight and both global, positive, and negative symptoms, but correlation coefficients were small and each of these domains explained only 7,2%, 6,3%, and 5,2% of the variance [7]. Since then, these associations have generally– but not unequivocally – been corroborated [8–11]. Conversely, in what is referred to as “the insight paradox”, good insight has been associated with increased levels of depressive symptoms and poorer quality of life [7, 12–14]. With regard to cognitive abilities, meta-analyses have shown a weak but statistically significant association between poor insight and several domains of social cognition, neurocognition, and IQ [10, 15]. Importantly, several studies have reported a consistent association between poor insight and pharmacological non-adherence [2, 16–18]. Meta-analyses have indicated that poor insight may indeed be amenable to treatment, but findings are not entirely consistent and reflect disparate approaches [19, 20]. Thus, empirical findings highlight the need for a more comprehensive understanding of the nature of poor insight into illness in schizophrenia.

Recent explanatory models of poor insight mostly focus on neuro-, social-, or meta-cognitive deficits [10, 21]. A complementary explanatory model comes from phenomenological psychopathology. This account, suggested by Henriksen and Parnas [22], proposes that impaired insight in schizophrenia, rather than a defect in cognitive processes, is tied to fundamental alterations of the structure of subjectivity, viz. self-disorders (a.k.a. disorder of basic self or basic self-disturbance). Self-disorders are associated with subsequent development of psychotic symptoms [23, 24] and persist temporally beyond state-psychopathology [25–27] (see [28, 29] for recent meta-analyses and a systematic review of the empirical literature). Such disorders entail a destabilization of “the natural ontological attitude”, i.e., our everyday, tacit experience of the world as real, mind-independent, and constrained by common-sense assumptions about space, time, causality and the principle of noncontradiction [30] (p.208). This usually unquestioned metaphysical framework allows our immersion in a shared social world that at the most basic level comes across to us as familiar, predictable, and ontologically secure (ontology concerns the fundamental nature, structure, and categories of being or existence) [1, 31]. A weakening of the natural ontological attitude in schizophrenia may allow a private-solipsistic (or autistic) experiential framework to emerge and, ungoverned by the certitudes of the natural ontological attitude, challenge common-sense assumptions about oneself, others, and the world [22]. Moreover, self-disorders are argued [22] to form the nucleus of the “felt” or immediately given experiential character of psychotic phenomena that infuses them with a sense of irrefutable subjective reality, often rendering full insight impossible [32, 33].

This study offers the first empirical examination of this phenomenological explanatory model of poor insight into illness in schizophrenia. Following this model, we hypothesized that self-disorders would be associated with poor clinical insight independently of shared associations with other forms of psychopathology.

## Methods

### Sample

The sample comprises 67 patients with a diagnosis of schizophrenia or non-affective psychosis (corresponding to the ICD-10 categories F20 and F22-29, see also Table 1), and it was combined from two independent, comprehensive psychopathological studies. The first study was a 5-year follow-up study of patients with first-admission status at baseline [26, 34]. The other study included patients with schizophrenia spectrum disorders in various stages of illness [35]. To be included in either study, patients had to be capable of tolerating lengthy interviews, which excluded aggressive, agitated, and/or severely psychotic patients. Additional exclusion criteria were primary or clinically dominating alcohol or substance use, organic brain disorder, mental retardation, involuntary admission, forensic status, and age > 65 years. The two aforementioned studies contributed, respectively, 35 and 32 participants to this sample.

**Table 1:** Descriptives of the sample

	Frequency (%) / Mean (SD)
N	67
Gender (F/M)	39/28
Age	30.3 (6.8); range: 19–52
Not working or studying	50 (75%)
Never married or cohabiting	34 (51%)
Diagnosis (ICD-10)	
Schizophrenia	59 (88%)
Delusional disorder	3 (4%)
Other nonorganic psychotic disorders	5 (7%)
Time since first psychotic symptom (months)	73.1 (80.1); median: 50; range:1-360
No. of inpatient admissions	3.3 (4.6); median: 2; range: 0–30
Positive symptoms (PANSS)	16.4 (4.9)
Negative symptoms (PANSS)	16.7 (5.7)
General symptoms (PANSS)	34.4 (7.6)
Functional level (GAF-F)	45.7 (13.4); Range: 25–80
Level of general intelligence	100.5 (10.8); range: 77–117
Medication	
Antidepressants	25 (37%)
Antipsychotics	37 (55%)

All individuals participated upon written consent, and the Danish Data Protection Agency approved the studies. According to Danish legislation, approval from The Danish National Committee on Health Research Ethics is not required for interview studies of this kind.

### Assessments and interviews

All patients were assessed for general psychopathology with a composite interview schedule used in several studies from our group [34, 36, 37]. The clinical interview included a thorough psychosocial history, a description of illness evolution, the Operational Criteria Checklist, OPCRIT [38] (created on the basis of the Present State Examination, PSE [39]), expanded with additional items from the Schedule for Affective Disorders and Schizophrenia, SADS-L [40], perceptual items from the Bonn Scale for the Assessment of Basic Symptoms, BSABS [41], a mental state examination targeting expressive features (e.g., affect modulation, stereotypies, mannerisms and formal thought disorder [42]), the PANSS [4], and the split version of the Global Assessment of Functioning, GAF [43]. General intelligence was assessed by a computerized test, Intelligenz-Struktur-Test 2000R [44], comprising verbal-, numerical, and figurative-spatial IQ represented by four selected subtests: analogies, sentence completion, sequences of numbers, and matrices. The results of these subtests were summed into a global IQ score used for data-analysis. Self-disorders were assessed with the Examination of Anomalous Self-Experience, EASE [45], which is a checklist for a semi-structured interview consisting of 57 main items. The EASE has demonstrated high internal consistency and inter-rater reliability [34, 46, 47] and is the gold-standard assessment for self-disorders. Insight was measured by the PANSS G12 item [15].

Interviews were conducted in a semi-structured, conversational way following phenomenological principles [1, 48, 49]. Rating an item was never based on a simple “yes” or “no” answer, but required examples of experiences described by the patients, which then were evaluated in accordance with the relevant item definitions. All interviews were conducted by experienced research clinicians (LSN and ARR) that had demonstrated good EASE interrater-reliability with JN, an official instructor and director of EASE courses (mean Cohen’s kappa 0,74 and 0,81). Like most previous EASE studies, scoring of EASE items was performed dichotomously, i.e., items were rated as ‘absent’ (including ‘absent’ and ‘doubtfully present’ items) or ‘present’ [28].

### Statistical analysis

We explored potential correlations between the PANSS G12 clinical insight measure and demographic variables with Mann-Whitney U-test and correlation analysis. Mann-Whitney U-test was used to compare scores on clinical scales and level of general intelligence in the groups with good insight (PANSS G12 rated as ‘absent’ or ‘minimal’) and impaired insight (‘mild’ or ‘moderate’). To investigate relations of the clinical dimensions of psychopathology with impaired insight, we first used simple linear regression with impaired insight (PANSS G12) as dependent variable and self-disorders (EASE total score), positive symptoms, negative symptoms, depressive symptoms (PANSS depression subscale, item G1, G2, G3, and G6 [50]), and general level of intelligence as independent variables. Subsequently, all independent variables were entered in a single step in a multiple linear regression model (*n* = 64 due to missing data regarding level of intelligence in three patients). We used SPSS version 25 for the analyses. Significance level was 0.05.

## Results

Sociodemographic and clinical characteristics are listed in Table 1. There were no significant associations between these variables and clinical insight. On the PANSS G12 item, 7 (10%) patients had “absence” of impairment of insight, 14 (20%) patients had “minimal” impairment of insight, 25 (37%) patients had “mild” impairment of insight, and 21 (31%) patients had “moderate” impairment of insight. On this basis, we divided the sample into two groups: one group with good insight (“absence” or “minimal” impairment of insight, *n* = 21) and one group with impaired insight (“mild” or “moderate” impairment of insight, *n* = 46). The group with impaired insight had a significantly higher level of self-disorders (mean EASE score 22.4 vs. 16.9; *p* = 0.01, Mann-Whitney U-test) and significantly higher level of general intelligence (mean score 102.6 vs. 96.0; *p* = 0.04, Mann-Whitney U-test) than the group with good insight. There were no significant group differences regarding positive, negative, or depressive symptoms.

In simple linear regression analysis, including these psychopathological dimensions and level of general intelligence as predictors, only self-disorders were significantly associated with impaired insight, although level of general intelligence was borderline significant (see Table 2). Subsequent multiple linear regression showed similar results (see Table 2).

**Table 2:** Linear regression analyses with impaired insight as outcome

	B	SE	β	T	*P*	*R* ^2^
Simple linear regression						
Self-disorders	0.034	0.014	0.281	2.359	0.021	0.079
Positive symptoms	0.003	0.025	0.013	0.104	0.918	< 0.001
Negative symptoms	-0.002	0.021	-0.012	-0.095	0.925	< 0.001
Depressive symptoms	-0.025	0.033	-0.096	-0.776	0.440	0.009
General intelligence	0.020	0.011	0.235	1.900	0.062	0.055
Multiple linear regression						0.154
Self-disorders	0.039	0.016	0.317	2.444	0.018	
Positive symptoms	0.001	0.035	0.001	0.005	0.996	
Negative symptoms	0.004	0.023	0.022	0.168	0.867	
Depressive symptoms	0.004	0.040	0.014	0.092	0.927	
General intelligence	0.024	0.012	0.285	1.971	0.054	

Note. *n* = 67. Yet *n* = 64 for general intelligence and multiple linear regression analysis

## Discussion

This study is the first to empirically examine the association between self-disorders and poor insight in schizophrenia and non-affective psychosis. The patients were in different stages of illness, but the majority had been ill for more than four years. Most had mild levels of current symptomatology and low occupational functioning. Level of insight varied between good and moderately impaired. We found that the group of study participants with impaired insight was characterized by a higher level of self-disorders and general intelligence than the group with good insight. On the other hand, we found no between-group differences concerning positive, negative, or depressive symptoms. In simple and multiple linear regression analyses, only self-disorders were significantly associated with impaired insight.

The original account, which motivated this empirical study, provided a different view on the nature of impaired insight in schizophrenia than the accounts dominating the literature [22]. According to the DSM-5, “unawareness of insight typically is a symptom of schizophrenia itself…… comparable to the lack of awareness of neurological deficits following brain damage, termed anosognosia” [51] (p.101). Contemporary research, though acknowledging the complexity of the problem, largely views poor insight as the manifestation of faulty information processing involving neuro-, social-, or meta-cognitive deficits [8]. As such, poor insight is construed as a rather circumscribed error of judgment or critical self-reflection on one’s own experiences and thinking. However, as argued by Henriksen and Parnas [22], these cognitive accounts presuppose an intact distinction between the symptoms of the illness and the self that evaluates these experiences. In other words, that it is in principle possible to conceptualize the notion of illness and its symptoms (e.g., rashes, polydipsia, and diabetes) as something that is distinct from the experiencing subject. If, in fact, schizophrenia is closely linked to a fundamental disorder of the self, involving “a variety of *alterations of the structures of experiencing*, affecting the very *conditions* of self-reflection” [22] (p. 543, original italics), these presuppositions become questionable. Thus, Henriksen and Parnas proposed that impaired insight in schizophrenia is not primarily cognitive in nature, but more fundamentally related to self-disorders.

In our empirical study, this explanatory model is supported by the association between poor clinical insight and self-disorders. Self-disorders involve a weakening of the first-personal manifestation (“mineness”) of experience, which is central to the experience of being a temporally stable, embodied and demarcated subject [32]. Here, we are dealing with subtle tears to the ordinary structure of subjectivity that usually allows us to feel naturally immersed in a fundamentally trustworthy, reliable, and predictable world, which is inhabited by others like us and exists independently of ourselves. Patients with such experiences may complain that they feel ephemeral, empty, ontologically different, and disconnected from their peers. They may explain how thought processes, emotions or bodily movements feel anonymous or estranged. Or they may report an inability to immediately grasp meaning and context, to let things be, and instead find themselves incessantly pondering aspects of their own inner life or the fabric of the intersubjective and physical world. Moreover, there may be an emergence of various solipsistic experiences that draw the most basic components of the self-world relation further into question. Patients may question the common sense existential and metaphysical frame of reference, including the mind-independent existence of the world and others, and the laws of physical reality. Instead, they may describe access to hidden layers or dimensions of reality, experiences of primary self-reference or fleeting feelings of centrality. Thus, self-disorders may lead to a weakening of the natural ontological attitude and the emergence of a private-solipsistic experiential framework.

Although self-disorders often involve affliction, patients usually also describe these as intrinsic and habitual aspects of their mode of experience and existence. For example, patients may be surprised that not all people experience their own thoughts as located to a specific part of the brain or with an acoustic quality. Empirical studies have shown that self-disorders typically precede the onset of psychosis [23], often being manifest already in childhood or early adolescence [24, 52], and tend to outlast the remission of frank psychosis [25–27]. However, the habitual character of self-disorders is not only related to their temporal persistence and early onset but also to fact that they affect the fundamental structure of subjectivity (first-personal manifestation), which is intrinsic to all our experiences [22].

The psychotic phenomena typical of schizophrenia, e.g. passivity phenomena [32, 53] and auditory hallucinations [54, 55], are closely linked with such disorders of subjectivity [56]. Moreover, the paradigmatic schizophrenic delusion, i.e., what Jaspers and Schneider dubbed “primary delusion” [1, 57, 58], originates as a revelation within the self, perhaps being preceded by a delusional mood. Here, the delusional meaning is revealed to the patient in a direct, unmediated manner as an irrefutable felt experience [22, 56]. Hence, the delusion is psychologically “incomprehensible”. Its content and mode of presentation is unrelated to inferences about empirical matters and, accordingly, the person rarely seeks supportive evidence in the external world. Rather, the absolute certainty of a primary delusion articulates itself passively within the subject’s immanent sphere. This “egological” nature [59, 60] of the primary delusion is perhaps comparable to the ordinary certainty of experiencing a sensation like pain or having a thought and makes complete insight difficult.

An important phenomenon, that adds to the complexity of the notion of insight, is the concept of double bookkeeping. Originally described by Bleuler [61], it refers to the ability of many schizophrenia spectrum patients to simultaneously inhabit and separate a psychotic world from the shared, social world [22, 33, 62]. Patients experience both worlds as relevant and real though not necessarily in the same sense of these terms [63–65], and although double bookkeeping may break down in acute psychotic exacerbations, most patients are, in a stable phase, able to distinguish these two worlds and to some extent keep them apart [62, 66]. Thus, a patient may agree with the clinician about the unreality of her hallucination and accept medication to relieve the affliction it causes, yet simultaneously preserve her conviction about the ultimate truth of the experience, e.g., as originating from another dimension of existence [67]. Here, the truth of the hallucinatory experience is not experienced as belonging to public, shared reality but as a distinct felt experience within subjectivity, and thus as “real” in another sense.

Given the main findings of recent meta-analyses outlined in the introduction, the absence of associations between impaired insight and positive, negative, or depressive symptoms in our study may seem surprising. This could be related to the fact that we examined patients in non-acute phases of illness. However, similar results have also previously been reported [68], and the present findings might also be seen to corroborate the fundamental assertion that poor insight is not just a reflection of symptom severity.

The weak positive association between higher general intelligence and poorer insight is also an unexpected finding. Most previous studies have reported a weak negative association between poor insight and general intelligence [10, 15]. The different findings may be related to the fact that studies examine insight in different phases of illness. In acute illness phase, a high level of general intelligence may improve some ability of self-correction, at least at a behavioral level and when confronted with an interviewer. We also speculate that patients with lower levels of intelligence may find it hard to articulate the double orientation, which, in our view, often characterizes a mild-to-moderate level of impaired insight. Typically, patients with this level of insight partially endorse the medical terms of psychiatry, while also articulating the incongruence of their experiences with the medical model.

A limitation of this study is the relatively small sample size. This, however, also reflects its main strength, namely its in-depth phenomenological interviews conducted by experienced research clinicians. Moreover, the sample does not include patients with severe impairment of insight. This is related to the recruitment, which for a large part of the sample depended on patients volunteering for a five-year follow-up assessment. Also, the lengthy interview schedule made it difficult to include patients in a severely disorganized or flamboyantly psychotic state. The sample has a high proportion of women, probably due to exclusion criteria that tended to exclude males. As cognitive performance was not the main focus of either of the original psychopathological studies, the Intelligenz-Struktur-Test 2000R was not employed *in toto*, and a history of specific learning disorders or a previous diagnosis of ADHD were not part of the exclusion criteria. Finally, as outlined in the introduction, insight is a complex phenomenon that may be conceptualized and measured in disparate ways. In this study we assessed clinical insight by means of the PANSS item G12. While this is a frequently used, valid, and reliable measure [15], it is of course less comprehensive than other widely used instruments like the SUMD [5].

## Conclusions

In sum, our findings lend preliminary support to the phenomenological model that suggested that impaired insight in schizophrenia is related to self-disorders. These habitual anomalous experiences destabilize the natural-ontological attitude and facilitate the emergence of a private-solipsistic framework for experiencing oneself, others, and the world. Moreover, they are closely related to the paradigmatic psychotic symptoms of schizophrenia and their sense of irrefutable subjective reality. Obviously, this explanatory model needs further empirical substantiation, but if corroborated, we believe it may have implications for early intervention and treatment. Thus, psychoeducational and psychotherapeutic approaches that attempt to increase insight and compliance may benefit from addressing self-disorders and their relation to psychotic symptomatology. In our view, it is of crucial importance not to dismiss patients’ experiences (self-disorders as well as psychotic symptoms) as mere cognitive errors or medical symptoms. Rather, clinicians must acknowledge their first-personal subjective reality and support the patient in negotiating a balance between a private-solipsistic experiential framework and the shared social world [33, 69].
